# Measuring Stigma in Pediatric Oncology: A Cross-Sectional Analysis of Three Global Sites

**DOI:** 10.1200/GO.24.00213

**Published:** 2025-01-08

**Authors:** Lara E. Counts, Robin S. Tanner, Yichen Chen, Meenakshi Devidas, Gia Ferrara, Inam Chitsike, Nester Chokwenda, Edith Matsikidze, Ana M. Cáceres-Serrano, Lucia Fuentes, Thelma Velasquez Herrera, Hadeel Halalsheh, Nadine Fraihat, Nickhill Bhakta, Sima Jeha, Victor M. Santana, Sara M. Malone, Dylan E. Graetz

**Affiliations:** ^1^ St Jude Children's Research Hospital, Memphis, TN; ^2^ University of Tennessee Health Science Center, Memphis, TN; ^3^ Parirenyatwa Hospital, Harare, Zimbabwe; ^4^ Unidad Nacional de Oncología Pediátrica, Guatemala City, Guatemala; ^5^ King Hussein Cancer Center, Amman, Jordan; ^6^ Washington University in St Louis School of Medicine, St Louis, MO

## Abstract

**PURPOSE:**

Stigma contributes to fear and shame, resulting in delays in care-seeking behavior among individuals with cancer. As a social construct, stigma is affected by language, religion, culture, and local norms. This study explored pediatric cancer stigma at the time of diagnosis across diverse settings through the adaptation of two stigma measures.

**METHODS:**

This study was conducted with adolescents and caregivers of children with osteosarcoma and retinoblastoma at three centers in Jordan, Guatemala, and Zimbabwe. The Stigma-related Social Problems (SSP) and the eight-item Stigma Scale for Chronic Illness (SSCI-8) measures were translated into Arabic, Spanish, and Shona and contextually adapted for use with adolescents and caregiver proxies. Adapted measures were pilot-tested and iteratively revised.

**RESULTS:**

Extensive adaptations were made to both measures to make them relevant to the local pediatric contexts. The final measures were used in nine patients and 28 caregivers. The exploratory analysis found that domain-specific and overall scale scores for both measures indicate a higher level of stigma than those found in previous studies (SSP: patient [51.23], caregiver [40.74]; SSCI-8: patient [50.41], caregiver [49.78]). Paired, patient-caregiver proxy responses were analyzed, with disagreement between the pairs for both scales.

**CONCLUSION:**

Adapted measures detected high levels of stigma among patients with pediatric cancer and their caregiver proxies and demonstrated a lack of concordance in the reports. This suggests the importance of studying stigma in this population and the need to ask patients about their stigma without using proxy measures. The required adaptations suggest a need for stigma measures developed specifically for pediatric cancer.

## INTRODUCTION

Stigma has been defined as “the co-occurrence of components of labeling, stereotyping, cognitive separation into ‘us’ and ‘them’ groups, status loss, social rejection, and discrimination, in the context of power differentials that allow one group to successfully devalue another.”^1^ Stigma has been identified as a barrier to health-seeking behavior, often associated with poor treatment adherence.^1-6^ Conceptualized dimensions of stigma include self-stigma or internalized stigma, enacted stigma, and public stigma. Enacted stigma refers to discriminatory behavior directed toward an individual because of a specific attribute, often leading to isolation and poor well-being. Internalized stigma, or self-stigma, occurs when a person learns stigmatizing ideas or stereotypes and applies them to themselves and has been especially noted as a contributing factor to treatment abandonment.^1,7-11^ Treatment abandonment, defined as up-front treatment refusal or a delay in therapy of 4 weeks or greater,^12^ leads to worse health outcomes and ultimately death in patients with pediatric cancer globally.^13^

**CONTEXT**

**Key Objective**
To adapt and use two stigma scales for patients with pediatric cancer and their caregiver proxies.
**Knowledge Generated**
Extensive adaptations were needed to use these stigma assessment instruments in Zimbabwe, Jordan, and Guatemala. Disagreement was found in paired patient-caregiver responses.
**Relevance**
Pediatric cancer stigma is a significant problem globally, and there is a need for a new measure that better assesses stigma to decrease treatment abandonment.

Since stigma is a social construct, it may be characterized differently across contexts.^14,15^ Common myths and misconceptions regarding pediatric cancer including those around causation, fatalism, contagion, blame, and changes to physical appearance may contribute to stigma.^16-18^ Local views, opinions, and beliefs affect stigma and may increase negative outcomes including treatment abandonment and worse quality of life.

While there is evidence suggesting that cultural factors affect the availability and use of cancer treatments,^18^ there is a lack of literature supporting the challenges of stigma in pediatric cancer across cultures. Validated stigma measures have been created for various health conditions.^2-5,19-25^ Some have been adapted for use in different languages and diverse cultural and geographic settings.^21-23^ None of these measures have been validated for use in pediatric cancer.^26,27^

The purpose of this study was to explore pediatric cancer stigma at the time of diagnosis across culturally and geographically diverse settings. Because of the lack of pediatric cancer–specific measures, we attempted to adapt and use two different measures: the Stigma-related Social Problems (SSP) scale and the eight-item Stigma Scale for Chronic Illness (SSCI-8), both of which have been used to assess cancer stigma among adults in various settings.^28-34^ These measures were translated into Arabic, Spanish, and Shona, adapted for local context, and used in Jordan, Guatemala, and Zimbabwe. These sites were selected due to the diversity in culture, language, and religion; additionally, selected as regions with an interest in how stigma affects cancer care.^35-37^

## METHODS

### Design

This cross-sectional multisite study was conducted in patients recently diagnosed with osteosarcoma and retinoblastoma and their caregivers. These two cancers were selected because of the appearance-altering surgeries that are often necessary and the high rates of treatment abandonment globally.^16-18^ This study was led by St Jude Children's Research Hospital in Memphis, TN, partnering with large pediatric cancer centers in Jordan (King Hussein Cancer Center [KHCC]), Guatemala (Unidad Nacional de Oncología Pediátrica [UNOP]), and Zimbabwe (Parirenyatwa Hospital). This study was part of a larger study that explored stigma through qualitative interviews (reported elsewhere).^38^ This study presents the adaptation and pilot testing of validated measures related to the stigma of health conditions.

### Setting and Participants

Selected sites differ in income levels, resource availability, religion, and culture. Jordan is a predominantly Muslim, upper-middle–income country where KHCC is a specialized cancer hospital. Guatemala, an upper-middle–income country located in Central America, has a predominantly Christian and Mayan population. UNOP is a pediatric cancer hospital. Zimbabwe, a country located in sub-Saharan Africa, is a culturally diverse, Christian Bantu, low-income country. Parirenyatwa Hospital is a university teaching hospital.^38^

Recruitment was conducted in each hospital by local research teams consisting of the site principle investigator and research assistants trained in survey administration. Participants included caregivers of children younger than 18 years with osteosarcoma or retinoblastoma and adolescent patients age 12-18 years. Recruiting caregiver-patient dyads was prioritized, when possible. All participants were newly diagnosed (within the 12 weeks before study enrollment) to allow for an assessment of up-front stigma as families transitioned from the community to the cancer center and to minimize recall bias.^38^ Participants spoke English, Arabic, Spanish, or Shona. The study teams attempted to enroll a socially and culturally diverse sample. Participants were compensated in accordance with local norms for their participation. An attempt was made to schedule study visits around regularly scheduled medical appointments to decrease burden on families.

### Ethics Statement

This study was reviewed and approved by the Institutional Review Board at St Jude Children's Research Hospital and by the regulatory boards responsible for human protection at each global site. Site PIs identified eligible participants, and a member of the local study team approached potential participants in person during a clinic visit or via phone call. Study teams verbally explained the study, and each participant provided informed consent in their native language in accordance with local policy. For minors, the consent was worded using language that was clear and understandable to children. Caregivers of minors provided consent for their participation, in accordance with local policies. Because of the sensitive nature of the interviews, psychosocial professionals were made available to all study participants.

### Measures

The two adapted and translated scales were administered verbally at the end of a semistructured qualitative interview.

#### SSP

The SSP intends to measure the psychosocial impact of various health conditions. In the original study, the SSP was created for a Swedish population with good validity and reliability (alpha = 0.90).^28^ The SSP includes 20 items divided into two domains: distress (10 items) and avoidance (10 items).^28^ The SSP has been further validated in a diverse adult oncologic population in Sweden.^29^ Each domain (distress and avoidance) has 10 items which were summed and transformed to 0-100 scales. Both domains were scored on a four-point Likert scale. The wording of the 10 items was same in each domain. The distress domain was scored from (1) definitely not bothered to (4) definitely bothered, whereas the avoidance domain was scored from (1) never to (4) almost always, that is, higher scores indicated greater dysfunction. Scores of <20 indicated no or very mild limitations, scores of 20-39 indicated mild impairment, scores of 40-59 indicated moderate impairment/limitations, and scores >59 indicated severe impairment/limitations.

#### SSCI-8

The SSCI-8 is a shortened version of the 24-item SSCI, a scale initially created to measure enacted and internalized stigma in adult neurological conditions.^30^ The SSCI-8 was created after psychometric testing of the full version suggestion item redundancy (alpha = 0.96), indicating that a shorter version would suffice.^30,31^ This tool was selected because of its use in chronic conditions, providing some disease similarity to cancers. The SSCI-8 has an enacted stigma domain (five items) and an internalized stigma domain (two items), with one item scoring in both domains. Reliability of the eight-item version was assessed at alpha = 0.89 in the original study.^31^ This scale has previously been validated or used in adult breast cancer populations in Iran, Hungary, and China.^32-34^ The SSCI-8 items were rated on a five-point Likert scale ranging from (1) never to (5) always, with a composite score ranging from 8 to 40 according to original tool guidance. Higher scores indicated higher levels of stigma. The enacted and internalized domains were summed to create a raw score ranging between 8 and 40. This raw score was rescaled to an Item Response Theory Scale^31^ that was standardized to 100, with a mean of 50 and a standard deviation of 10.

To our knowledge, neither measure has been validated or used in pediatric cancer settings.

### Procedures

#### Adaptation and Piloting

The local teams in Jordan, Guatemala, and Zimbabwe reviewed the SSP and SSCI-8 in English and adapted the measures on the basis of local context. This included removal and rewording of some items to localize the tools (changes described in the Results section). A professional company translated the scales to Arabic, Spanish, and Shona.^38^ The translated interview guide was reviewed by the research team and piloted with patients and caregivers at each site. Pilot testing was conducted verbally in each respective language. Revisions to the interview guide related to item wording and order were made according to feedback informally elicited in pilot testing. The interview guides were then back-translated to English by bilingual members of the study team to maintain the accuracy of the measure. A final reconciliation of the scales was conducted, and the interview guide was finalized.

#### Data Collection

Scales were administered verbally in the respective languages by local members of the study team to patients and caregivers as proxies.^38^ The adapted SSP and SSCI-8 scales were scored for patients and caregivers using paper forms. Data were subsequently entered into Qualtrics by research associates at St Jude Children's Research Hospital.^39^

#### Data Analysis

Individual-level stigma scores and demographic information were summarized using descriptive statistics. When applicable, paired patient-caregiver responses were analyzed descriptively. Both the SSP and SSCI-8 items were scored and analyzed consistent with original tool guidance, described above. A score was calculated if a respondent answered at least half of the items. An exploratory analysis investigated stigma across study sites.

## RESULTS

### Scale Adaptation and Cognitive Interviewing

Through review with local teams and piloting with 12 caregivers and seven patients, both the SSP and SSCI-8 were adapted for use with local populations. Complete changes to the scales are provided in Data Supplement (Tables S1 and S2). No patients or caregivers declined participation in the pilot. Recruitment is presented in Data Supplement (Table S3).

The SSP was adapted in multiple ways. A question regarding physical intimacy was deemed inappropriate for a pediatric population and removed. The SSP was further adapted for specific social contexts at each site. In Zimbabwe, adolescents do not go to restaurants, so the item of “Going to restaurants” was changed to “Going to weddings or parties (eating).” Similarly, “Traveling by public transportation” was changed to “Traveling,” “Trying on and buying clothes” to “Getting dressed for a special event,” and “Bathing/Swimming in public places” to “Changing clothes in public area to participate in an event.” In Guatemala and Zimbabwe, “Going to the cinema or theatre” was changed to “Going to church.”

Both scales were intended for use with both the patient and the caregiver, with wording to indicate either you/your child. After piloting, participants in Zimbabwe recommended splitting the Shona questionnaire into separate versions for the patients and caregivers. No other changes were made to the SSCI-8.

### Characteristics

This study included 37 participants: nine patients and 28 caregivers. Across the three sites, only six participants who were approached declined participation. The median age of patients was 15 years (IQR, 13-16.7). Most were males (n = 6), diagnosed with osteosarcoma (n = 8), with localized disease (n = 7). Most patients' treatment plans included surgery (n = 9) and chemotherapy (n = 7). Caregivers included 23 mothers, four fathers, and one grandparent. The median age of caregivers was 37.5 years (IQR, 29.5-45.5). Thirteen caregivers had a child diagnosed with osteosarcoma, whereas 15 caregivers had a child with retinoblastoma. Most caregivers were married (n = 22). Native language and religion varied by site. Characteristics of participants are presented in Tables 1 and 2.

**TABLE 1:** Patient Characteristics

Patient	Guatemala	Jordan	Zimbabwe	Total
Median age, years	13.5 (IQR, 12-15)	15.75 (IQR, 15-16.67)	14.5 (IQR, 12-17)	15 (IQR, 13-16.67)
Diagnosis	2	5	2	9
Retinoblastoma	—	—	1	1
Osteosarcoma	2	5	1	8
Sex	2	5	2	9
Male	2	3	1	6
Female	0	2	1	3
Extent of disease	2	5	2	9
Localized	2	4	1	7
Metastatic	—	1	1	2
Treatment plan	3	10	5	18
Chemotherapy	1	5	1	7
Surgery	2	5	2	9
Radiation	—	—	1	1
No cancer-directed therapy (palliation only)	—	—	1	1

**TABLE 2:** Caregiver Characteristics

Caregiver	Guatemala	Jordan	Zimbabwe	Total
Median age, years	35.5 (IQR, 30-38)	40 (IQR, 28-46)	39.5 (IQR, 34.5-45)	37.5 (IQR, 29.5-45.5)
Child's diagnosis	10	10	8	28
Retinoblastoma	5	5	5	15
Osteosarcoma	5	5	3	13
Child's sex	10	10	8	28
Male	5	6	5	16
Female	5	4	3	12
Relationship	10	10	8	28
Mother	9	7	7	23
Father	1	3	—	4
Grandparent	—	—	1	1
Marital status	10	10	9	29
Married	5	10	7	22
Separated/divorced	1	—	1	2
Widowed	1	—	1	2
Living together, not legally married	3	—	—	3
Religion	11	10	8	29
Islam	—	10	—	10
Christian	8	—	6	14
Catholic	1	—	2	3
None	2	—	—	2
Native language	12	10	8	30
Arabic	—	10	—	10
Shona	—	—	8	8
Spanish	9	—	—	9
Indigenous language	3	—	—	3

### Scales

#### SSP

Scores were calculated overall and for each domain, and exploratory analyses are presented by site and across centers (Tables 3 and 4).

**TABLE 3:** SSP Scale

Participant	No.	Mean (SD)	Min	Q25	Median	Q75	Max
Patient Jordan	5	62.7 (12.82)	50	52.78	58.33	75	77.78
Caregiver Jordan	9	48.77 (19.3)	27.72	38.89	47.22	50	94.44
Patient Guatemala	2	36.11 (11.79)	27.78	31.94	36.11	40.28	44.44
Caregiver Guatemala	10	29.72 (10.31)	13.89	25.69	31.94	33.33	47.22
Patient Zimbabwe	2	37.50 (17.68)	25	31.25	37.50	43.75	50
Caregiver Zimbabwe	8	45.49 (18.36)	27.78	36.81	38.89	47.92	80.56
Patient total	9	51.23 (18.06)	25	44.44	50	58.33	77.78
Caregiver total	27	40.74 (17.84)	13.89	30.56	38.89	47.22	94.44

NOTE. Scores ranged from 0 to 100 with high scores related to a higher level of psychosocial impact.

Abbreviations: SD, standard deviation; SSP, Stigma-related Social Problems.

**TABLE 4:** SSP Scores

Participant	No or Very Mild Limitations	Mild Impairment	Moderate Impairment	Severe Impairment	Total
Patient Jordan	—	0	3	2	5
Caregiver Jordan	0	4	4	1	9
Patient Guatemala	—	1	1	0	2
Caregiver Guatemala	2	7	1	0	10
Patient Zimbabwe	—	1	1	0	2
Caregiver Zimbabwe	0	5	1	2	8
Patient total	—	2	5	2	9
Caregiver total	2	16	6	3	27

Abbreviation: SSP, Stigma-related Social Problems.

##### Distress.

The median distress score for patients across sites was 50 (range, 25-77.8). Median distress scores for patients in Jordan, Guatemala, and Zimbabwe were 50 (range, 50-77.8), 36.1 (range, 27.8-44.4), and 37.5 (range, 25-50), respectively. The scores of two patients indicated mild impairment, five indicated moderate impairment, and two indicated severe impairment. The median distress score for caregivers was 38.9 (range, 13.9-94.4). Median distress scores for caregivers in Jordan, Guatemala, and Zimbabwe were 47.2 (range, 27.8-94.4), 31.9 (range, 13.9-47.2), and 38.9 (range, 27.8-80.6), respectively. Two caregiver scores indicated no or very mild impairment, 16 indicated mild impairment, six indicated moderate impairment, and three indicated severe impairment.

##### Avoidance.

The median avoidance score for patients was 55.6 (range, 38.9-75). Median patient avoidance scores for Jordan, Guatemala, and Zimbabwe were 63.9 (range, 38.9-75), 55.6 (range, 52.8-58.3), and 52.8 (range, 50-55.6), respectively. One patient score indicated mild limitations, five indicated moderate limitations, and three indicated severe limitations. The median avoidance score of all caregivers was 41.7 (range, 16.7-80.6). Mean scores for Jordan, Guatemala, and Zimbabwe were 47.2 (range, 30.1-75), 44.4 (range, 16.7-80.6), and 33.3 (range, 19.4-69.4), respectively. Two caregiver scores indicated no or very mild limitations, 11 indicated mild limitations, eight indicated moderate limitations, and five indicated severe limitations.

#### SSCI-8

Scores were calculated overall and for each domain, and exploratory analyses are presented by site and across centers (Table 5).

**TABLE 5:** SSCI-8

Participant	No.	Mean (SD)	Min	Q25	Median	Q75	Max	ES Mean (SD)	ES Range	IS Mean (SD)	IS Range
Patient Jordan	4	47.345 (6.07)	39.20	45.50	48.45	50.40	53.70	25 (3.33)	20-26.67	36.67 (13.88)	20-53.33
Caregiver Jordan	9	46.43 (4.62)	39.20	45.70	47.60	50.60	51.70	24.07 (4.94)	20-33.33	28.15 (10.94)	20-53.33
Patient Guatemala	2	54.15 (0.64)	53.70	53.93	54.15	54.38	54.60	41.67 (2.36)	40-43.33	33.33 (18.86)	20-46.67
Caregiver Guatemala	10	49.34 (9.79)	39.20	39.20	50.20	58.20	35.20	40 (23.88)	20-86.67	29.33 (14.47)	6.67-53.33
Patient Zimbabwe	2	52.60 (2.83)	50.60	51.60	52.60	53.60	54.60	40 (9.43)	36.67-43.33	30 (4.71)	26.67-33.33
Caregiver Zimbabwe	8	54.10 (4.25)	47.60	51.92	54.55	56.40	60.80	42.50 (13.06)	26.67-60	40.83 (16.11)	13.33-60
Patient total	8	50.41 (5.23)	39.20	48.87	52.15	53.93	54.60	32.90 (9.50)	20-30	34.17 (12.50)	20-53.33
Caregiver total	27	49.78 (7.37)	39.20	45.70	50.60	54.55	63.20	35.43 (17.86)	20-86.67	32.35 (14.52)	20-60

Abbreviations: ES, enacted stigma; IS, internalized stigma; SD, standard deviation; SSCI-8, eight-item Stigma Scale for Chronic Illness.

##### Enacted.

The median enacted stigma scores of patients across sites was 30 (range, 20-46.7). Enacted stigma scores from Guatemala and Zimbabwe were similar, with respective medians of 41.7 (range, 40-43.3) and 40 (range, 33.3-46.7). The median domain score for Jordan was lower: 26.7 (range, 20-26.7). The median domain score of caregivers across sites was 30 (range, 20-86.7). Site domain scores among caregivers in Jordan, Guatemala, and Zimbabwe varied, with medians of 23.3 (range, 20-33.3), 28.3 (range, 20-86.7), and 41.7 (range, 26.7-60), respectively.

##### Internalized.

The median internalized stigma score of patients was 33.3 (range, 20-53.3). Median domain scores for Jordan, Guatemala, and Zimbabwe were 36.7 (range, 20-53.3), 33.3 (range, 20-46.7), and 30 (range, 26.7-33.3), respectively. The median internalized stigma score for caregivers was 33.3, with a wide range from 6.7 to 60. Domain scores among caregivers in Jordan, Guatemala, and Zimbabwe varied, with medians of 26.7 (range, 20-53.3), 26.7 (range, 6.7-53.3), and 46.7 (range, 13.3-60), respectively.

#### Paired Responses

**FIG 1:**
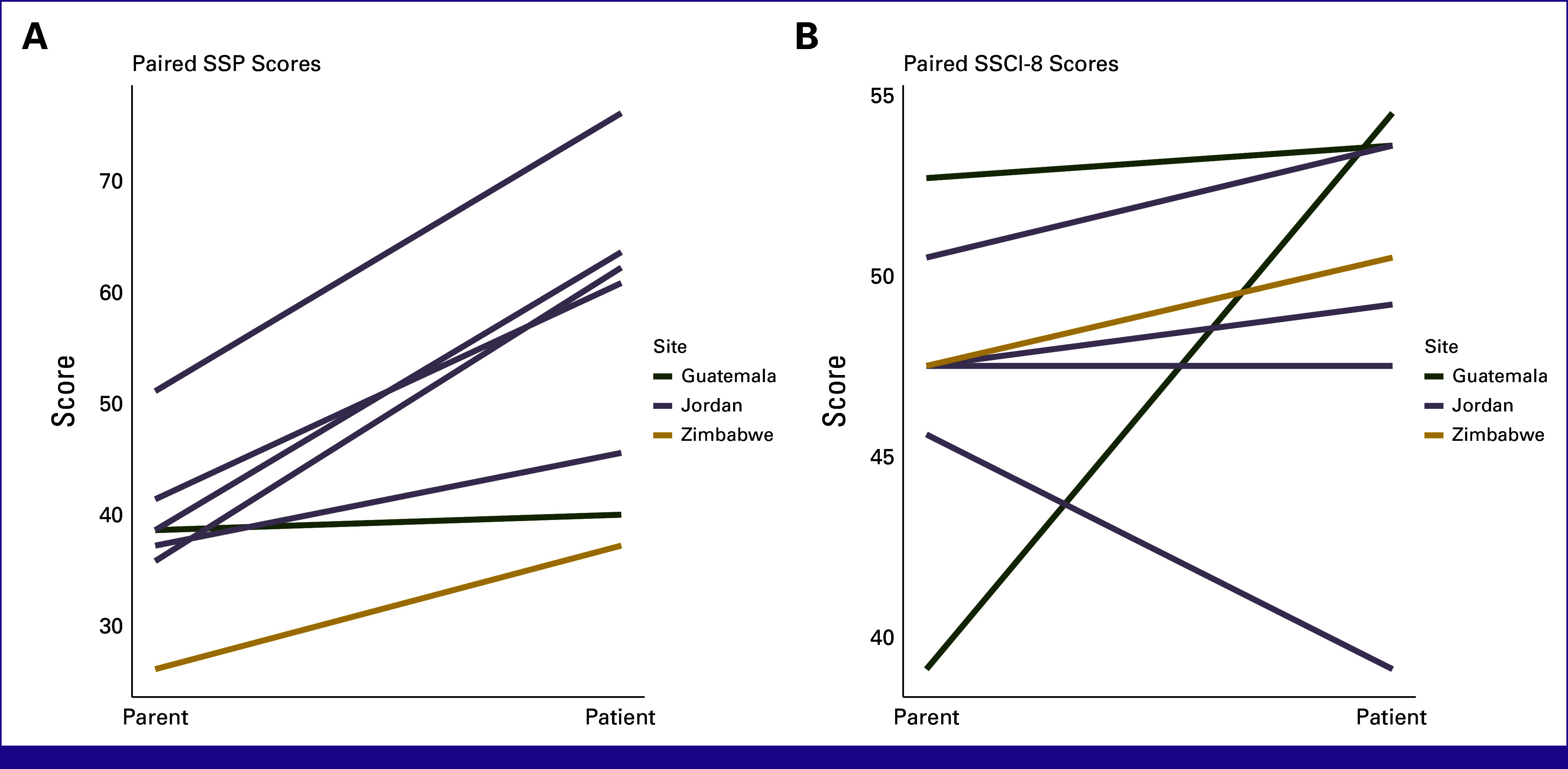
(A) SSP and (B) SSCI-8 paired responses. SSCI-8, eight-item Stigma Scale for Chronic Illness; SSP, Stigma-related Social Problems.

Exploratory analysis was conducted on eight patient-caregiver dyad responses, with a total of 16 opportunities for agreement per measure (Fig 1). Summary scores for each measure were matched and compared. Disagreement was defined as a score difference of 5 or greater, after review of previous publications of these tools.^31^ When observing stigma across the SSP, there was dyad disagreement 11 times (of 15, one not applicable [NA]), with 10 patients reporting higher stigma than their caregiver. For overall SSP scores, seven patients reported higher stigma scores than their caregiver proxy, with a range of 1.39-26.39 and an average difference of 16.67 points. On the SSCI-8, there was disagreement among the pairs in 8 of 14 scores (two NA).

## DISCUSSION

We noted high levels of stigma in patients with pediatric cancer and caregivers across diverse settings. These results suggest that stigma is present from the time of diagnosis and has been understudied among children with cancer globally. Because of the cultural and ethnic diversity of participants in this study, extensive adaptations to the measures were required. After these adaptations, our study found higher levels of stigma than in the previously published literature, across both measures. This may suggest higher levels of stigma in this population than those previously studied. Alternatively, it is possible that differences were due to adaptations and translations, which affected face validity of the tools.

In the original Swedish population that piloted the SSP, the mean distress and avoidance scores were 25.3 and 21, respectively.^28^ In our study, average distress scores were much higher, at 51.2 for patients and 40.1 for caregivers. Average avoidance scores were also higher, at 58 for patients and 44.8 for caregivers. Our adapted SSCI-8 also demonstrates much higher stigma levels than in previous studies.^40-42^ One study on pain captured a mean stigma score of 21,^40^ whereas an MS study captured a mean stigma score of 12.^41^ In our study, patient stigma scored 50.4, whereas caregiver stigma scored 49.8; these high scores illustrate that enacted and internalized stigmas are an immediate concern for children and caregivers of children with cancer.

Jordan had the highest overall SSP scores but the lowest overall SSCI-8 scores. Recent studies including adult participants in Jordan demonstrate that Jordanians have positive attitudes about cancer and toward patients with cancer.^43^ The low scores on the SSCI could be extrapolated to suggest lower levels of stigma in the Jordanian population, but this makes our SSP results from Jordan, which suggest high levels of distress and avoidance related to stigma, surprising. Our previous qualitative work has found that Jordanians often do not report cancer as being a contagious disease, whereas participants from Guatemala and Zimbabwe do. Both the SSP and SSCI-8 have questions related to contagion; however, it is possible that the SSCI-8 has more questions perceived to be due to contagion, resulting in a lower overall SSCI-8.^38^ Alternatively, it could be that while Jordanians experience and thus report lower levels of enacted or internalized stigma, the distress and avoidance that result are higher in this population. Finally, it is possible that these discrepancies are related to adaptations altering scale validity or our small sample size.

With the exception of the avoidance subscale in Zimbabwe, patients rated stigma higher than their caregivers on the SSP, whereas caregiver and patient SSCI-8 scores are more similar. This is relevant because across disease states, many studies use proxy measures, in which it is expected that caregivers score similar to their children.^44^ Results from this study highlight how caregivers may be poor proxies for patient stigma. This could be due to caregiver's experience of associative stigma, making it difficult for them to report stigma as proxy. Future work should explore the validity of proxy measurement and the utility of a specific tool for caregivers focused on associative stigma.

This study should be considered in light of several limitations. Data were collected at the time of diagnosis, and future work is needed to assess stigma throughout the cancer continuum. Verbal administration of the survey might have led to response bias. In addition, the sample size was small, and thus, all findings are exploratory. Future work with larger sample sizes is needed for development of a valid and reliable stigma measure for pediatric cancer that can be used globally. Finally, development of a separate tool to assess associative stigma experienced by caregivers is warranted.

In conclusion, our findings indicate that stigma may be highly present in patients with pediatric cancer and caregivers across Jordan, Guatemala, and Zimbabwe. Stigma is a complex, multicomponent phenomenon, and locally adapted measures may not adequately assess stigma in these regions. It is necessary to ensure that there are scales that comprehensively assess pediatric cancer stigma. Future work is needed to develop measures that will enable a thorough and specific assessment of stigma in children with cancer and their caregivers globally.

## Data Availability

Data are available at reasonable request from the authors.

## References

[1] Link BG, Phelan JC: Conceptualizing stigma. Annu Rev Sociol 27:363-385, 2001

[2] Earnshaw VA, Quinn DM: The impact of stigma in healthcare on people living with chronic illnesses. J Health Psychol 17:157-168, 2012 21799078 10.1177/1359105311414952 PMC8919040

[3] Earnshaw VA, Quinn DM, Park CL: Anticipated stigma and quality of life among people living with chronic illnesses. Chronic Illn 8:79-88, 2012 22080524 10.1177/1742395311429393 PMC3644808

[4] Thornicroft G: Stigma and discrimination limit access to mental health care. Epidemiol Psichiatr Soc 17:14-19, 2008 18444452 10.1017/s1121189x00002621

[5] Wetterneck C, Memorial HR: Stigma and shame as barriers to treatment in obsessive-compulsive and related disorders. J Depress Anxiety 4:3, 2015

[6] Arora RS, Eden T, Pizer B: The problem of treatment abandonment in children from developing countries with cancer. Pediatr Blood Cancer 49:941-946, 2007 17252565 10.1002/pbc.21127

[7] Corrigan P, Watson A: The paradox of self-stigma and mental illness. Clin Psychol Sci Pract 9:35-53, 2002

[8] Corrigan PW, Druss BG, Perlick DA: The impact of mental illness stigma on seeking and participating in mental health care. Psychol Sci Public Interest 15:37-70, 2014 26171956 10.1177/1529100614531398

[9] Catona D, Greene K, Magsamen-Conrad K, et al: Perceived and experienced stigma among people living with HIV: Examining the role of prior stigmatization on reasons for and against future disclosures. J Appl Commun Res 44:136-155, 2016

[10] Boyle MP: Enacted stigma and felt stigma experienced by adults who stutter. J Commun Disord 73:50-61, 2018 29574262 10.1016/j.jcomdis.2018.03.004

[11] Corrigan PW, Rao D: On the self-stigma of mental illness: Stages, disclosure, and strategies for change. Can J Psychiatry 57:464-469, 2012 22854028 10.1177/070674371205700804 PMC3610943

[12] Weaver MS, Arora RS, Howard SC, et al: A practical approach to reporting treatment abandonment in pediatric chronic conditions. Pediatr Blood Cancer 62:565-570, 2015 25586157 10.1002/pbc.25403

[13] Weaver MS, Howard SC, Lam CG: Defining and distinguishing treatment abandonment in patients with cancer. J Pediatr Hematol Oncol 37:252-256, 2015 25757024 10.1097/MPH.0000000000000319

[14] Yang LH, Kleinman A, Link BG, et al: Culture and stigma: Adding moral experience to stigma theory. Soc Sci Med 64:1524-1535, 2007 17188411 10.1016/j.socscimed.2006.11.013

[15] Surbone A: Cultural aspects of communication in cancer care. Support Care Cancer 16:235-240, 2008 18196291 10.1007/s00520-007-0366-0

[16] Graetz D, Rivas S, Fuentes L, et al: The evolution of parents’ beliefs about childhood cancer during diagnostic communication: A qualitative study in Guatemala. BMJ Glob Health 6:e004653, 2021 10.1136/bmjgh-2020-004653 PMC8160167 34039587

[17] Krishnan Y, V Sankar U, Gazel S, et al: Public perception on childhood cancers from a population-based study in South India: Lessons to learn to avoid stigma. Pediatr Hematol Oncol J 8:242-246, 2023

[18] Daher M: Cultural beliefs and values in cancer patients. Ann Oncol 23:66-69, 2012 22628419 10.1093/annonc/mds091

[19] Yilmaz M, Dissiz G, Usluoǧlu A, et al: Cancer-related stigma and depression in cancer patients in a middle-income country. Asia Pac J Oncol Nurs 7:95-102, 2020 31879690 10.4103/apjon.apjon_45_19 PMC6927157

[20] Huang Z, Yu T, Wu S, et al: Correlates of stigma for patients with cancer: A systematic review and meta-analysis. Support Care Cancer 29:1195-1203, 2021 32951087 10.1007/s00520-020-05780-8

[21] Pallavi P, Bakhla AK, Akhouri PK, et al: Stigma scale adaptation and validation for measuring COVID-19 stigma. Cureus 15:e38744, 2023 37303349 10.7759/cureus.38744 PMC10247905

[22] Huang F, Chen WT, Shiu CS, et al: Adaptation and validation of a culturally adapted HIV stigma scale in Myanmar. BMC Public Health 21:1663, 2021 34517850 10.1186/s12889-021-11685-w PMC8439000

[23] Bahar Z, Cal A, Beser A, et al: A study on the adaptation of the HIV/AIDS-related Stigma Scale into Turkish. Perspect Psychiatr Care 58:509-517, 2022 34878644 10.1111/ppc.12984

[24] Golberstein E, Eisenberg D, Gollust S: Perceived stigma and mental health care seeking. Psychiatr Serv 59:392-399, 2008 18378838 10.1176/ps.2008.59.4.392

[25] Quinn DM, Chaudoir SR: Living with a concealable stigmatized identity: The impact of anticipated stigma, centrality, salience, and cultural stigma on psychological distress and health. J Pers Soc Psychol 97:634-651, 2009 19785483 10.1037/a0015815 PMC4511710

[26] Horan MR, Sim JA, Krull KR, et al: A review of patient-reported outcome measures in childhood cancer. Children 9:1497, 2022 36291433 10.3390/children9101497 PMC9601091

[27] Gavan L, Hartog K, Koppenol-Gonzalez GV, et al: Assessing stigma in low- and middle-income countries: A systematic review of scales used with children and adolescents. Soc Sci Med 307:115121, 2022 35843180 10.1016/j.socscimed.2022.115121

[28] Ohlsson-Nevo E, Karlsson J: Impact of health-related stigma on psychosocial functioning in the general population: Construct validity and Swedish reference data for the Stigma-related Social Problems scale (SSP). Res Nurs Health 42:72-81, 2019 30499114 10.1002/nur.21924 PMC6827347

[29] Ohlsson-Nevo E, Ahlgren J, Karlsson J: Impact of health-related stigma on psychosocial functioning in cancer patients: Construct validity of the Stigma-related Social Problems scale. Eur J Cancer Care (Engl) 29:e13312, 2020 32865867 10.1111/ecc.13312 PMC7757179

[30] Rao D, Choi SW, Victorson D, et al: Measuring stigma across neurological conditions: The development of the Stigma Scale for Chronic Illness (SSCI). Qual Life Res 18:585-595, 2009 19396572 10.1007/s11136-009-9475-1 PMC2875076

[31] Molina Y, Choi SW, Cella D, et al: The Stigma Scale for Chronic Illnesses 8-item version (SSCI-8): Development, validation and use across neurological conditions. Int J Behav Med 20:450-460, 2013 22639392 10.1007/s12529-012-9243-4 PMC3758464

[32] Daryaafzoon M, Amini-Tehrani M, Zohrevandi Z, et al: Translation and factor analysis of the Stigma Scale for Chronic Illnesses 8-item version among Iranian women with breast cancer. Asian Pac J Cancer Prev 21:449-455, 2020 32102523 10.31557/APJCP.2020.21.2.449 PMC7332131

[33] Vizin G, Szekeres T, Juhász A, et al: The role of stigma and depression in the reduced adherence among young breast cancer patients in Hungary. BMC Psychol 11:319, 2023 37814282 10.1186/s40359-023-01355-4 PMC10561463

[34] Jiang N, Zhang YX, Zhao J, et al: The mediator role of stigma in the association of mindfulness and social engagement among breast cancer survivors in China. Support Care Cancer 30:5007-5015, 2022 35192056 10.1007/s00520-022-06882-1 PMC8861258

[35] Borges AA, de Lima RAG, Dupas G: Secrets and truths in the process of family communication with a child with cancer. Escola Anna Nery 20:1-9, 2016

[36] Arabiat DH, Alqaissi NM, Hamdan-Mansour AM: Children’s knowledge of cancer diagnosis and treatment: Jordanian mothers’ perceptions and satisfaction with the process. Int Nurs Rev 58:443-449, 2011 22092322 10.1111/j.1466-7657.2011.00899.x

[37] Walubita M, Sikateyo B, Zulu JM: Challenges for health care providers, parents and patients who face a child hood cancer diagnosis in Zambia. BMC Health Serv Res 18:314, 2018 29720168 10.1186/s12913-018-3127-5 PMC5932785

[38] Graetz DE, Velasquez T, Chitsike I, et al: Stigma in pediatric cancer: An exploratory study of osteosarcoma and retinoblastoma in Guatemala, Jordan, and Zimbabwe. JCO Glob Oncol 10:e2400017, 2024 38905576 10.1200/GO.24.00017 PMC11191872

[39] Qualtrics. [computer program] Version 2022. Provo, UT, Qualtrics, 2005

[40] Scott W, Yu L, Patel S, et al: Measuring stigma in chronic pain: Preliminary investigation of instrument psychometrics, correlates, and magnitude of change in a prospective cohort attending interdisciplinary treatment. J Pain 20:1164-1175, 2019 30940501 10.1016/j.jpain.2019.03.011

[41] Maurino J, Martínez-Ginés ML, García-Domínguez JM, et al: Workplace difficulties, health-related quality of life, and perception of stigma from the perspective of patients with multiple sclerosis. Mult Scler Relat Disord 41:102046, 2020 32179482 10.1016/j.msard.2020.102046

[42] Meca-Lallana JE, Prefasi D, Pérez-Miralles F, et al: Perception of stigma in patients with neuromyelitis optica spectrum disorder. Patient Prefer Adherence 15:713-719, 2021 33880015 10.2147/PPA.S305707 PMC8052114

[43] Al Qadire M, Khaldoun AM, Alsrayheen E, et al: Public attitudes toward cancer and cancer patients: A Jordanian national online survey. Middle East J Cancer 13:352-362, 2022

[44] Forman SM, Connolly ME, Hardy SJ: Reliability of parent proxy-report measures of quality of life and cognitive functioning in pediatric sickle cell disease. Blood 140:10881-10882, 2022

